# A case of recurrent RESLES type I: analysis of pathogenic factors and delayed imaging manifestations

**DOI:** 10.3389/fmed.2026.1815607

**Published:** 2026-04-10

**Authors:** Zhihui Yu, Zhongxun He, Kang Du, Daijun Zhang, Hongzhi Wan, Mengting Shi, Haohao Wu, Yunli Yu

**Affiliations:** 1 Department of Neurology, Affiliated Hospital of Guizhou Medical University, Guiyang, Guizhou, China; 2 Department of Neurology, Yunnan Qujing Central Hospital (Qujing First People’s Hospital), Qujing, Yunnan, China

**Keywords:** high recurrence, immunotherapy, MRI delayed effect, predisposing factors, RESLES

## Abstract

Reversible splenial lesion syndrome (RESLES) is a clinico-radiological syndrome characterized by a reversible lesion in the splenium of the corpus callosum. It is more common in children, and most patients recover completely with active treatment. However, recurrent RESLES is very rare and its etiology is unknown, posing significant challenges for clinicians in terms of prediction, diagnosis, and treatment. This article reports the first case of RESLES Type I in a young adult with five episodes and identifiable predisposing factors (intracranial infection and immunosuppressant dependence). By analyzing the patient’s medical history, neuroimaging characteristics, body fluid test results, and response to immunomodulatory therapy, and reviewing recent literature on recurrent RESLES. The findings suggest that intracranial infection and immune dysfunction may be potential factors leading to the recurrence of RESLES Type I in this case, while serum sodium levels can remain within the normal range. Furthermore, we observed that infe cranial MRI lesions associated with RESLES in this patient often appeared 4–7 days after symptom onset, indicating a potential delay in imaging changes. Therefore, it is recommended that clinicians select appropriate timing for neuroimaging examinations and consider repeat scanning when necessary to reduce the risk of misdiagnosis or missed diagnosis.

## Introduction

Reversible splenial lesion syndrome (RESLES) is a clinico-radiological syndrome manifesting as an isolated lesion in the splenium of the corpus callosum or more extensive lesions, accompanied by encephalopathic symptoms (1). Reversibility and self-limitation are its core features. Based on whether the lesion is confined to the splenium of the corpus callosum, RESLES can be classified into two types: Type I lesions are restricted to the splenium, while Type II involves extra-splenial brain regions (2). Although RESLES is rare and its mechanism remains unclear, the overall prognosis is good, with most patients experiencing a monophasic course and rapid resolution (3). However, in recent years, some even rarer cases of recurrent RESLES have attracted clinical attention, and the exploration of its recurrence mechanism and risk factors has been a focus scholars are eager to summarize (4). It was previously believed (4, 5) that recurrent RESLES still carries a good prognosis, with most patients experiencing only 1–2 recurrences, and clinical symptoms and imaging lesions resolving quickly with active treatment. This article reports a case of RESLES Type I in a patient who experienced five episodes, aiming to analyze the associated recurrent factors and neuroimaging characteristics to enhance clinicians’ understanding of this condition.

## Case report

**FIGURE 1:**
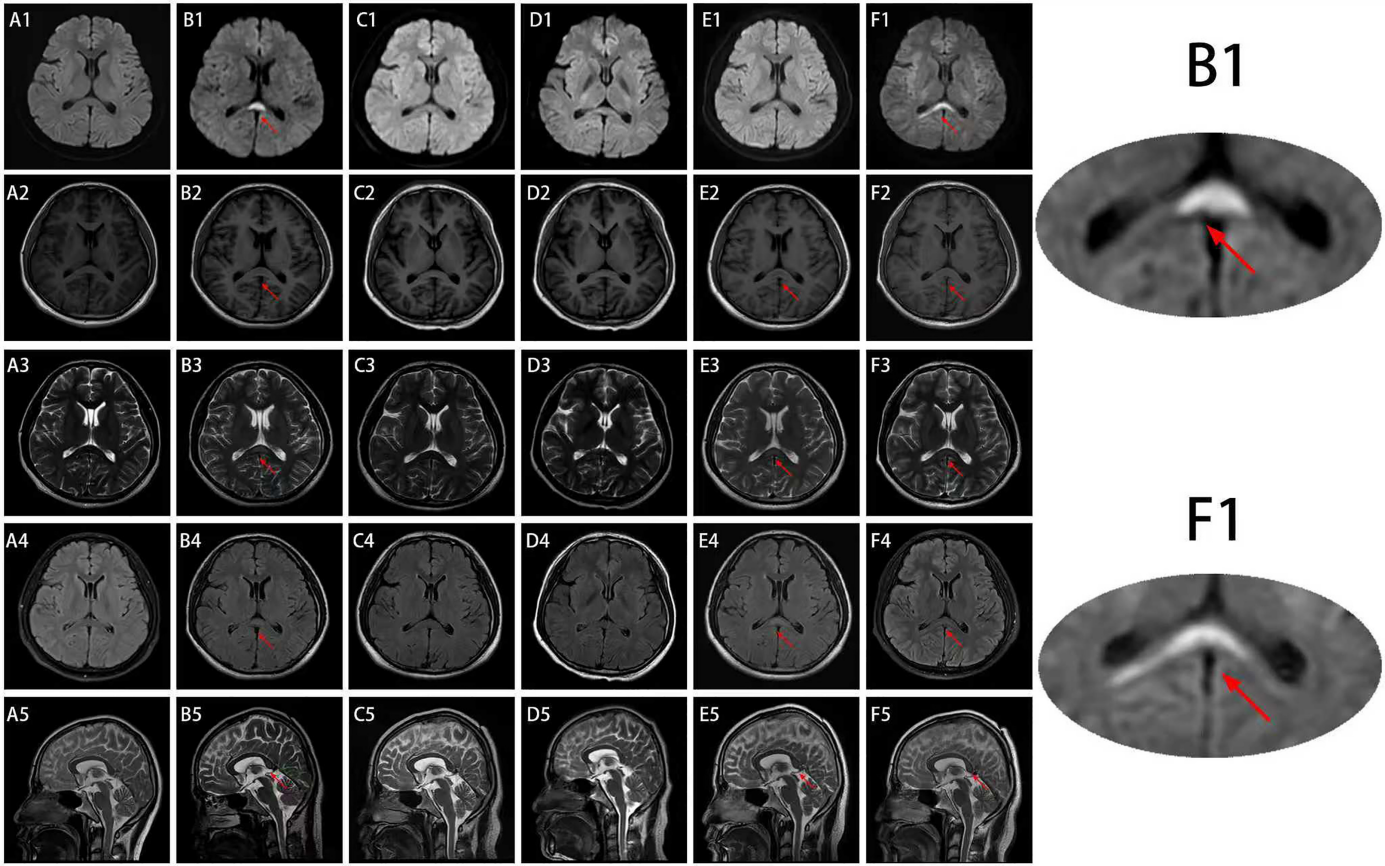
Brain Magnetic Resonance Imaging (MRI) findings of the patient during five episodes. **(A2–5)** No significant hyperintensity on brain MRI on day 8 after the first episode. **(B1–5)** Brain MRI on day 5 after the second episode showing hyperintensity (red arrow). **(C1–5)** The abnormal signal disappeared after 1 month of re-examination. **(D1–5)** No significant abnormalities on brain MRI on day 4 after the third episode. **(E1)** No significant abnormalities on DWI on day 4 after the fourth episode; T1WI, T2WI, and FLAIR **(E2–5)** on day 7 showing hyperintensity in the splenium of the corpus callosum. **(F1–5)** Abnormal signals in the splenium of the corpus callosum on day 4, **(A1)** the abnormal signal disappeared after 8 months. [**(A1–F1)**: DWI, **(A2–F2)**: T1WI; **(A3–F3)** and **(A5–F5)**: T2WI; **(A4–F4)**: FLAIR].

A 26-year-old male patient (as of 2 April 2018) developed cold symptoms such as cough, sputum production, and runny nose after catching a chill. Suspected community-acquired pneumonia, he was treated with oral antibiotics, which showed no improvement. Subsequently, he developed fever, persistent headache, confusion, and generalized headache, manifesting as delayed response, incoherent speech, and agitation, accompanied by a drunken gait. He experienced one generalized tonic-clonic seizure on the same day, which resolved spontaneously after several minutes. The patient had no significant past medical history or personal history, and there was no family history of similar diseases. Cerebrospinal fluid (CSF) examination suggested intracranial infection, but cranial Magnetic Resonance Imaging (MRI) and Electroencephalography (EEG) performed 8 days after onset showed no significant abnormalities. He was treated with antibiotics, mannitol, acyclovir, ganciclovir, and other medications, and his symptoms had alleviated at discharge (Table 1 and Figure 1). Over the next nearly 7 years, the patient experienced a total of four recurrences (30 May 2020; 9 December 2020; 24 November 2021; and 13 February 2025). Each episode was preceded by a “cold or catching a cold,” followed by varying degrees of headache, dizziness, and cognitive and behavioral disturbances. A seizure occurred again during the last recurrence, with the same pattern and duration as before. The final neurological examination revealed decreased calculation ability and memory, slowed response, clumsiness in rapid alternating movements of both upper limbs, unsteadiness in the finger-to-nose test and bilateral heel-to-shin test, inability to walk in a straight line, and a positive Romberg sign. CSF examination during each recurrence indicated intracranial infection, but tests for autoimmune encephalitis antibodies, demyelinating antibody profiles, and oligoclonal bands were negative. EEG mostly showed normal results or non-specific increased slow waves (Table 1).

**TABLE 1:** Clinical information, selected laboratory findings, and treatment regimens during the five episodes in the RESLES patient.

Time of onset	Clinical manifestations	CSF	EEG	Serum Na^+^ (mmol/L)	Treatment
2018-04-02	Headache, dizziness, mental/cognitive/ consciousness disturbances, ataxia, seizure	Pressure 300 mmH_2_O, clear, TCC 180 × 10^6^/L, NCC 5 × 10^6^/L, TP 490 mg/L, Cl^–^ 132.6 mmol/L, TORCH: (−)	Normal	140.30	Ganciclovir 350 mg Q12 h, acyclovir 0.5 g Q8 h, ceftriaxone 2 g Q12 h.
2020-05-29	Headache, dizziness, mental/cognitive/ consciousness disturbances, ataxia	Pressure 125 mmH_2_O, clear, WBC 10 × 10^6^/L, TP 642 mg/L, IL-6 18.41 pg/mL, TORCH: RuV-IgG (+), HSV2-IgG (+), CMV-IgG (+), AE related antibodies (−)	Normal	144.03	Methylprednisolone 120 mg daily, acyclovir 0.5 g Q8 h
2020-12-09	Headache, dizziness, mental/cognitive/ consciousness disturbances, ataxia	Pressure 210 mmH_2_O, clear, WBC16 × 10^6^/L, TP 556 mg/L, IL-6 4.16 pg/mL, TORCH: (−), AE related antibodies (−)	Focal slow-wave activity in the temporal region	143.03	Methylprednisolone 250 mg daily, acyclovir 0.5 g Q8 h, mycophenolate mofetil 0.5 g twice daily
2021-11-24	Headache, dizziness, mental/cognitive/ consciousness disturbances, ataxia	Pressure 180 mmH_2_O, clear, WBC30 × 10^6^/L, TP 1,289 mg/L, IL-6 8.98 pg/mL, TORCH:RuV-IgG (+), AE related antibodies (−), oligoclonal bands and demyelinating antibodies (−)	Normal	144.27	Methylprednisolone 500 mg daily, mycophenolate mofetil 0.75 g twice daily
2025-02-13	Headache, dizziness, mental/cognitive/ consciousness disturbances, ataxia, seizure	Pressure 185 mmH_2_O, clear, WBC13 × 10^6^/L, TP 586 mg/L, IL-6 6.20 pg/mL, TORCH: (−)	Diffuse slow-wave activity increased over all leads	144.65	Methylprednisolone 500 mg daily, mycophenolate mofetil 0.5 g titrated up to 0.75 g twice daily, sodium valproate 0.5 g twice daily

AE, autoimmune encephalitis; CSF, cerebrospinal fluid; EEG, electroencephalogram; IL-6, interleukin-6; NCC, nucleated cell count; RESLES, reversible splenial lesion syndrome; TCC, total cell count; TP, total protein; WBC, white blood cell.

Cranial MRI consistently revealed a “boomerang sign” in the splenium of the corpus callosum, most prominent on DWI, ADC, and FLAIR sequences (except for the 1st and 3rd episodes), leading to a diagnosis of RESLES Type I (2). Notably, a consistent temporal pattern was observed across multiple recurrences: neuroimaging findings were negative in the early disease stage, with lesions appearing later. Specifically, during the 2nd, 3rd, 4th, and 5th episodes, cranial MRI performed within 1–3 days of symptom onset revealed no causative lesions. However, during the 2nd (Figure 1, B1–5), 4th (Figure 1, E2–5), and 5th (Figure 1, F1–5) episodes, lesions became detectable on MRI examinations conducted 4–7 days post-onset. It is important to note that the timing of MRI scans was not entirely consistent across all five episodes. For instance, during the first episode, only one MRI was performed on day 8, which showed negative findings (Figure 1, A2–5). The absence of interim imaging data precludes determination of whether a lesion might have transiently appeared and completely resolved prior to the day-8 scan. Meanwhile, serum sodium levels remained within the normal range during all five episodes, with specific values of 140.30, 144.03, 143.03, 144.27, and 144.65 mmol/L, respectively (reference range 137–147 mmol/L, Table 1). The CSF interleukin-6 (IL-6) levels were elevated above the normal range during the 2nd, 4th, and 5th episodes, measuring 18.41, 8.98, and 6.20 pg/mL, respectively (reference range 0–4.4 pg/mL). This test was not performed during the 1st episode, and the result was normal during the 2nd episode (Table 1).

Regarding treatment, the patient received acyclovir combined with methylprednisolone pulse therapy during the 2nd (June 2020) and 3rd (December 2020) episodes, achieving a marked short-term therapeutic effect. Considering the recurrence within a mere 4-month interval, which suggested potential underlying immune dysfunction, immunosuppressive therapy was initiated following the third episode with mycophenolate mofetil 0.5 g twice daily. Nearly a year later, the patient experienced the fourth episode. After treatment with methylprednisolone pulse therapy and an increase in mycophenolate mofetil to 0.75 g twice daily, the patient remained recurrence-free for nearly 3 years. Subsequently, a fifth recurrence occurred during dose reduction (February 2025). The acute phase was again managed with methylprednisolone pulse therapy, resulting in significant symptom improvement. During the maintenance phase, mycophenolate mofetil was restored to the previously effective dose, and valproic acid was added for seizure control (Table 1). No recurrence was observed during a 7-month follow-up, and a follow-up DWI scan confirmed the resolution of the abnormal lesion from the fifth episode (Figure 1,A1).

## Discussion

Reversible splenial lesion syndrome was initially discovered by Tada et al. and named Mild Encephalitis/Encephalopathy with a Reversible Splenial Lesion (MERS). Garcia-Monco et al. systematically elaborated on MERS based on previous research and ultimately proposed the definition of RESLES as a broad term encompassing various etiologies. Both terms are still used interchangeably in the literature; this article uniformly uses RESLES for description (1, 6, 7). The main clinical features of RESLES include transient, self-limiting encephalopathic symptoms such as psychiatric and behavioral abnormalities, seizures, dizziness, and ataxia. Based on imaging, it can be classified into Type I, involving only the splenium of the corpus callosum, and Type II, involving the entire corpus callosum or even extensive white matter. Lesions are most prominent as high signal on DWI and FLAIR sequences and low signal on ADC maps. They typically resolve within 1 week, generally not exceeding 2 months, showing a locked-time relationship with clinical symptoms (3, 8). Due to the relative rarity of the disease, epidemiological data are limited, with most reports originating from Asian regions such as Japan and China (7, 9). A previous epidemiological study on acute encephalopathy in children from Japan indicated that RESLES (termed MERS in that study) ranked second among all pediatric acute encephalopathy syndromes, accounting for approximately 16% of cases (10). With increasing clinical recognition of the disease, the number of newly diagnosed cases annually shows an upward trend (11). This patient exhibited typical imaging findings confined to the splenium of the corpus callosum (Figure 1) and common clinical symptoms, thus being diagnosed with RESLES Type I. However, a young adult patient with RESLES Type I experiencing as many as five episodes over 7 years has not been reported in the literature worldwide. To our knowledge, only one pediatric patient with RESLES Type II has been reported to have experienced five episodes (4). Therefore, a detailed analysis of this patient from the perspectives of pathogenesis or predisposing factors, imaging characteristics, and treatment response is necessary.

RESLES is associated with various predisposing factors, among which infection is the most common. The mechanism may be related to certain viral infections activating inflammatory factors and oxidative stress, inducing neurotoxicity (12, 13). This patient had a “cold or catching a cold” before each episode, multiple CSF examinations revealed abnormally elevated levels of IL-6 (18.41, 8.98, and 6.20 pg/mL during the 2nd, 4th, and 5th episodes, respectively; reference range 0–4.4 pg/mL). IL-6, a pro-inflammatory cytokine, can promote Th17 cell differentiation, inhibit Treg cell function, disrupt blood-brain barrier integrity, and potentially enhance autoimmune responses (14). Similar observations of elevated IL-6 have been reported in other case reports of the same type, leading to the hypothesis that pro-inflammatory cytokines may be involved in the pathogenesis of RESLES (15). Therefore, we speculate that a chronic, easily relapsed underlying immunopathological mechanism might exist in certain susceptible individuals, possibly involving the synergistic effect of pathogen-triggered abnormal immune activation, blood-brain barrier dysfunction, and the production of specific autoantibodies, leading to recurrent reversible inflammatory edematous lesions in the splenium of the corpus callosum (12, 16). Although this patient’s CSF autoantibody tests for autoimmune encephalitis were negative, this does not preclude the possibility of other autoantibodies or T-cell-mediated immune abnormalities (17). It is worth noting that whether IL-6 abnormality can serve as a biomarker for RESLES recurrence requires further study. Furthermore, compared to the most common hyponatremia in RESLES (7), it is noteworthy that this patient’s serum sodium level was within the normal range during each episode. Xue et al. reported that 50% (3/6) of recurrent RESLES cases had normal sodium levels (4). The above findings suggest that the phenomenon of normal serum sodium may exist in patients with recurrent RESLES, but this finding has so far only been observed in sporadic case reports and still requires further validation with more clinical data.

Another noteworthy point is that during this patient’s five episodes, cranial MRI showed no significant abnormalities within 1–3 days, while lesions appeared 4–7 days after onset. This evolution and delayed effect in imaging have not been reported in the literature, nor reports on the optimal imaging time window for RESLES. However, due to the limitations of irregular scan timing across the five episodes, it is difficult to infer the optimal diagnostic window based on dynamic imaging changes. Nevertheless, based on the imaging evolution in this patient, we suggest considering days 4–7 after onset as a key time window for MRI scanning to improve the positive detection rate. Concurrently, we hypothesize that clinical symptoms may precede the appearance of imaging lesions. If MRI examination is delayed, characteristic lesions could be missed as they might have already resolved, further underscoring the necessity for repeated MRI scans in such patients. More systematic and intensive imaging follow-up in the future is required to further elucidate the dynamic evolution of RESLES lesions.

Currently, there are no standardized treatment guidelines for RESLES; management primarily consists of symptomatic and supportive care (18). Presently, methylprednisolone and intravenous immunoglobulin can be administered to patients with RESLES, alongside selective treatment based on etiology (7). In this case, the patient received antiviral therapy with acyclovir and methylprednisolone pulse therapy during the acute phase, resulting in rapid symptom alleviation, which aligns with the treatment efficacy reported in previous literature for the acute phase (7, 15, 18). However, after adding mycophenolate mofetil for immunomodulatory therapy, although the recurrence interval was significantly prolonged, relapses still occurred at a dose of 0.5 g twice daily. The control effect was excellent after increasing the dose to 0.75 g twice daily, but relapse occurred again during the dose reduction process, indicating a dose-dependent characteristic of the immunosuppressant in this patient, which might also be one of the factors contributing to his long-term recurrence.

## Conclusion

This study reports a case of RESLES Type I with five episodes. Combined with a literature review, we observed that in this case, intracranial infection and immunoregulatory dysfunction may be associated with the frequent recurrence and prolonged course of RESLES Type I, and the serum sodium levels could remain within the normal range. Furthermore, cranial MRI revealed characteristics of delayed lesion appearance and rapid resolution, suggesting a potential lack of clear temporal correlation between imaging changes and clinical symptoms. Clinicians should be alert to this phenomenon to avoid misdiagnosis. In terms of treatment, active intervention in the acute phase in this case was effective in controlling episodes, and long-term immunosuppressive therapy might reduce the risk of recurrence. This suggests that for patients with frequent recurrences, management as a chronic condition with long-term, close follow-up may be necessary. It should be noted that the above observations are based on a single case and cannot lead to generalized conclusions. This article aims to provide references for a deeper understanding of RESLES, and the related findings still require verification through subsequent follow-up and more clinical data.

## Data Availability

The raw data supporting the conclusions of this article will be made available by the authors, without undue reservation.
